# Executive decision support for managing AI risk in public health

**DOI:** 10.3389/ijph.2026.1610326

**Published:** 2026-10-26

**Authors:** Olusimbo Ige, Dolapo Fakuade

**Affiliations:** 1 Chicago Department of Public Health, Chicago, IL, United States; 2 School of Environment, Coventry University, Coventry, United Kingdom; 3 Emerging Technologies and Artificial Intelligence (ETAI) Centre, Khazar University, Azerbaijan

**Keywords:** AI governance, AI in public health, AI risk managament, decion making, public health leadership

## Abstract

Artificial Intelligence (AI) is transforming public health decision-making, shifting it from descriptive epidemiological reporting to predictive, accelerated operational guidance. Yet AI in public health is discussed almost entirely at technical or regulatory levels, leaving a gap in executive-level guidance. Public health operates within a complex landscape of social determinants, political scrutiny, and incomplete information, where algorithmic errors can severely damage public trust. Consequently, executive competency must expand beyond traditional data interpretation to foundational AI literacy. Leaders must differentiate among diverse AI applications and evaluate their risk profiles, including balancing AI trained on individual-level precision medicine models with public health population-level prevention mandate. Continuous human oversight and multidisciplinary validation should be standard for modern public health organizations. Automated systems must equitably advance population health outcomes under final human authority at all times. Public health executives can better prepare to make informed decisions about AI-related risks using the decision points presented in this commentary.

## Introduction

Artificial Intelligence (AI) is increasingly used to enhance public health functions with applications spanning disease surveillance, health resource planning, population risk stratification, outbreak prediction, and health policy evaluation [1–4]. While AI enables faster analysis of large datasets and more timely responses to health threats, the population-level impact of public health decisions requires due diligence to equitably advance population health outcomes [5]. The public health operational context is often characterized by incomplete information, political scrutiny, competing priorities, and complex interplay of social determinants. In such a context, algorithmic bias or inaccurate predictions can severely damage public trust [6]. AI must therefore be viewed not just as a technical tool, but as a major component of executive decision-making. As AI integrates into public health practice, managing its risks should become a core executive responsibility, not a delegated task to technical specialists. This commentary argues that public health leaders need a better understanding of how to balance technical evidence and human judgement. To assist leaders facing these high-consequence decisions, we propose a decision framework outlined in Table 1.

**TABLE 1:** Executive decision framework for responsible AI adoption in public health agencies (Chicago, USA, 2026).

Domain	Executive questions for review	Potential consequences if unaddressed
Public health fit and intended use	Is AI necessary and appropriate for this public health decision? Is the intended use aligned with a population-level prevention objective rather than an individual-level clinical prediction or treatment objective?	Deploying tools built for the wrong paradigm (e.g., precision-medicine models applied to population surveillance)
Data, validity, and local relevance	1. What data were used to develop, train, validate, and locally test the model? Are the data relevant, complete, current, and representative of populations served?	Models that underperform for marginalized or under-represented groups
Explainability and documentation	Can the organization explain, in appropriate terms to decision makers and communities, how the system generates its recommendation? Is there documented traceability for data sources, model development or procurement, versioning, validation, deployment approvals, and changes after launch?	Opaque “black box” decisions that cannot withstand public or political scrutiny
Equity, bias, and access	How is bias assessed and monitored? What is the anticipated equity impact, including any risk of restricting access to services?	Discriminatory outcomes; erosion of public trust
Governance & approval	Who is accountable for approval, implementation, escalation, and suspension or withdrawal of the system? Has an accountable executive formally approved the use case, risk controls, and conditions for deployment??	Diffusion of accountability; high-consequence decisions made without executive sign-off
Legal, regulatory, privacy, and security	What applicable state, federal, local, contractual, and international requirements apply? What is the plan for tracking a fast-evolving policy landscape?	Non-compliance; legal and reputational exposure
Operational readiness and implementation	Does the agency have the workforce capacity, data infrastructure, workflow integration, training, vendor support, and incident-response processes required to use the system safely?	Failed implementation; inappropriate reliance on outputs; workflow disruption; inability to identify, investigate, or correct harms
Monitoring, evaluation, and human oversight	What is the plan for ongoing monitoring of accuracy, drift, and bias? Who reviews outputs for errors after implementation, and who makes final decisions to override, pause, or discontinue the system?	Undetected model degradation; unsafe automation without human oversight

## Why AI is changing executive decisions

In public health, executives are no longer asking whether AI will become important. They are wrestling with whether the organization has the data, governance, infrastructure, and workforce to operationalize AI safely. Historically, public health leaders bridged the “data-to-action gap” by translating descriptive data such as a 12% increase in overdose mortality into strategic actions. Today, AI-enabled epidemiology can directly integrate surveillance, intervention effectiveness, and geospatial patterns to offer predictive and prescriptive intelligence to executives. For instance, an AI tool can identify high-burden communities and estimate how scaling specific outreach programs will prevent a precise number of deaths. These predictive efficiencies have driven AI adoption [5]. However, the assumptions and trade-offs connecting epidemiological observations to recommended actions often remain concealed. Transparency is critical because public health choices are rarely based solely on mathematical predictions; they demand thorough consideration of ethics, equity, and political context. Previously, decision makers depended on epidemiologists to convert raw data into actionable insights. Now, with AI processing both surveillance and operational data, executives must transition from operational inquiries to addressing ethical and policy-related questions required to guide high-consequence decisions [5, 7].

## AI literacy as an executive competency

Before making organizational decisions, executives must understand when and why AI was used, how decisions are reached, and what safeguards are in place. To evaluate responses, public health executives need a foundational understanding of the underlying technology. AI is not a single tool, but a collection of distinct approaches designed to improve efficiency. Machine Learning (ML) identifies patterns within historical data to generate classifications while Predictive AI forecasts future events like disease outbreaks. Generative AI (GenAI) creates new content, including reports and text summaries. Computer vision processes visual data like radiological images for automated surveillance. Recently, AI agents have emerged as autonomous systems capable of executing multi-step tasks, retrieving information across platforms, and coordinating workflows under human oversight [5]. Because these technologies have different functionalities and risk profiles, AI literacy is no longer a niche technical skill; it has become an essential core competency for contemporary public health leadership.

## When is AI the right tool?

To address complex public health issues like drug overdoses, executives could leverage predictive AI. Conventional approaches cannot match AI’s speed and scale in real-time disease surveillance, outbreak forecasting, and health system optimization. AI can rapidly analyze large datasets, generate tailored recommendations, provide evidence to guide resource allocation, and model consequences of alternative interventions. However, leaders must recognize the tension between population-level and individual-level risk. Most commercial AI is built for individual risk prediction (precision medicine), differing from public health’s focus on populations and prevention. Public health focuses on collective risk identified through population-wide surveillance. Leaders must recognize that AI built for hospitals is not automatically suited for public health workflows.

## Explainability, documentation, and regulations

Next is the AI explainability challenge. Many high-performing models are hard to explain to policymakers, practitioners, or affected communities. Trust hinges on how clearly a system can explain itself, making AI explainability a core leadership responsibility. Leaders must ensure model transparency and guarantee data quality, relevance, completeness, and representativeness. If an overdose prediction model is trained primarily on insured populations, it will underperform in marginalized communities. Executives must enforce strict documentation and traceability, vetting what data was used, how models were built, how decisions were generated, and who approved deployment.

The regulatory landscape is shifting rapidly. In 2026, over 43 US states introduced AI bills targeting chatbot oversight and clinical disclosure requirements [8]. The Centers for Medicare & Medicaid Services (CMS) opened rulemaking on AI-powered fraud detection [9], while the European Union AI Act established a risk-based framework classifying most population-health AI as high-risk [10]. Navigating this patchwork requires strong governance. Data security, interoperability, and privacy compliance cannot be delegated to algorithms.

## Evaluating risk: efficiency vs. acceptability

Executives must look beyond AI’s efficiency to assess acceptability. Leaders must evaluate how biases are monitored and how health equity is impacted. For tools impacting entire populations, executives must define unacceptable risk. Following EU AI Act guidelines, AI must not discriminate or restrict access to services [10]. This requires caution and governance when deploying AI for risk prediction determining resource allocation or program eligibility. Chatbots and virtual assistance could be lower risk with transparency and defined data sources. Minimal-risk applications include spam filters and scheduling tools with few regulatory requirements.

## Monitoring and evaluation as a safeguard

AI in public health requires continuous human oversight. Epidemiologists must review outbreak alerts, clinicians must validate high-risk recommendations, and leaders must retain final decision authority. Mandatory monitoring and evaluation (M&E) provide mechanisms to assess safety, equity, and real-world performance against population health goals. Organizations must monitor model drift, accuracy declines, and algorithmic bias over time. A model predicting overdoses accurately today may fail if demographics shift. Mitigating risks requires multidisciplinary input from epidemiologists, data scientists, legal counsel, and equity experts. AI recommendations should be validated against observed results so corrective action can be taken. The core principle: do not deploy AI until comprehensively reviewed for vulnerabilities.

### Conclusion

Public health decision-making is not merely technical. Leaders need not know how code works, but must verify what data trained in the AI, who is accountable when it errs, and whether the system can explain outputs. Transparent, disciplined AI governance is now the primary differentiator for modern public health organizations. Table 1 operationalizes these principles.
